# Development of experimental silicosis in inbred and outbred mice depends on instillation volume

**DOI:** 10.1038/s41598-019-50725-9

**Published:** 2019-10-02

**Authors:** Jessica M. Mayeux, Dwight H. Kono, Kenneth Michael Pollard

**Affiliations:** 1 Department of Molecular Medicine, The Scripps Research Institute, 10550 North Torrey Pines Road, La Jolla, CA 92037 USA; 2 Department of Immunology and Microbiology, The Scripps Research Institute, 10550 North Torrey Pines Road, La Jolla, CA 92037 USA

**Keywords:** Animal disease models, Respiration

## Abstract

There is considerable variation in methods to induce experimental silicosis with the effects of dose and route of exposure being well documented. However, to what extent the volume of silica suspension alters the dispersion and severity of silicosis has not been adequately investigated. In this study, the optimal volume of a crystalline silica suspension required to obtain uniform distribution and greatest incidence and severity of silicosis was determined in inbred and outbred mice. Silica dispersal, detected by co-inspiration with India ink and polarized light microscopy, was highly dependent upon volume. Furthermore, although peribronchitis, perivasculitis, and increases in bronchoalveolar lavage fluid cell numbers were detected a lower doses and volumes, significant alveolitis required exposure to 5 mg of silica in 50 μl. This dose and volume of transoral instillation led to a greater penetrance of silicosis in the genetically heterogeneous Diversity Outbred strain as well as greater alveolar inflammation typical of the silicosis in human disease. These findings underscore the critical importance of instillation volume on the induction, severity, and type of inflammatory pathology in experimental silicosis.

## Introduction

Silica exposure is a well-known occupational hazard for individuals working in the dusty trades and can result in the fibrotic lung disease silicosis^1–3^. Inhalation of silica containing dust leads to chronic inflammation of the lung, with development of silicosis being dependent upon the cumulative dose of silica exposure^4–8^. This is supported by animal model studies, which have shown a direct correlation between dose and severity of lung disease^9,10^. The presentation and severity of disease also depends on the genetic predisposition with DBA/2, MRL/MpJ, NZB, NZM2410, (NZBxNZW)F1, and C57BL/6 being more sensitive than C3H/HeN, BALB/c, and CBA/J^9–13^. Experimental induction of silicosis also depends on the form of silica^1,14^ and the route of exposure^14–16^.

There is currently no accepted standard experimental protocol for the exposure of mice to silica or the induction of silicosis and pathological sequelae such as autoimmunity. A wide range of approaches have been reported including inhalation of aerosolized silica using either whole body or head/nose-only exposures^3^, or instillation of silica suspension via intranasal (IN)^16–18^, intratracheal (IT)^15,16,19–23^, or transoral/oropharyngeal (TO) routes^11,14–16,24–26^. Inhalation of aerosolized particles most closely model human exposure^3^, however these procedures require specialized equipment, use large amounts of potentially valuable material, and require repeated exposures over lengthy time periods^3,14,15^. Instillation of silica in a liquid carrier, most often phosphate buffered saline (PBS), can be less wasteful of valuable reagents, requires less costly apparatus, but can also be technically challenging. IN instillation is the easiest method to administer particulate material such as crystalline silica, however it produces less severe disease than either IT or TO routes^16,27^. IT and TO instillation also allows reproducible delivery of material to the lungs in a short time period and avoids exposure to skin or pelt which is an issue with whole body inhalation^28^. However, the IT technique is technically challenging and invasive as it requires instilling silica directly into the lumen of the trachea either by use of a catheter or needle, or by surgically exposing the trachea (also called transtracheal instillation)^28^. The non-surgical catheter approach does not have the potential issues of recovery from surgery and the complication of unwanted inflammation^15^ but is best suited to species where the mouth can be opened enough to view the vocal cords^28^. The TO route has been shown to distribute silica evenly and more uniformly than the IT surgical technique, with less variability and more pronounced alveolitis^15^. Furthermore, the TO route was superior for inducing other respiratory diseases such as asthma^29^ and bleomycin induced fibrosis^30^. Moreover, a greater percentage of radiolabeled submicrometer colloidal suspension was detected in the lung after IT (77%) or TO (62–81%, 25–50 μls respectively) routes compared with aerosolized (32%) delivery, with the percentage in the lung following TO instillation found to be volume dependent^31^. Thus, the TO approach has several advantages over other methods of inducing silicosis.

Although previous studies have noted the role of dose, time, mouse strain and instillation route on the severity of experimental silicosis^9–11,15,16^, little attention has been given to the volume of material instilled. While several volumes, notably 1–2 ml/kg (50–100 μls/20 gram mouse)^28^ and 3 μl/gram of body weight^32^, have been recommended for IT instillation of particulate material, these were not based on studies using the TO route or crystalline silica for the induction of silicosis.

Initial experiments using TO instillation of Evans Blue dye showed that dye dispersal was dependent upon volume and lung lobe size. We hypothesized that larger volumes of a crystalline silica containing solution would similarly enhance distribution of particles to the periphery of the lungs leading to more widespread inflammation particularly of the alveoli. To test this, we used the TO route to instill different doses and volumes of crystalline silica and assessed subsequent lung pathology in inbred and outbred mice. We determined that the volume of administration affects the severity of silicosis and that mice receiving the same dose in a larger volume have more diffuse inflammation and more severe pathology. This was especially true for genetically heterogeneous Diversity Outbred (DO) mice where the larger volume led to greater disease incidence. We conclude that TO instillation of silica requires an appropriate volume for the induction of diffuse inflammation and severe experimental silicosis, particularly in a genetically heterogeneous population.

## Results

### Effect of instillation volume on pulmonary dispersion of instilled fluid

**Figure 1:**
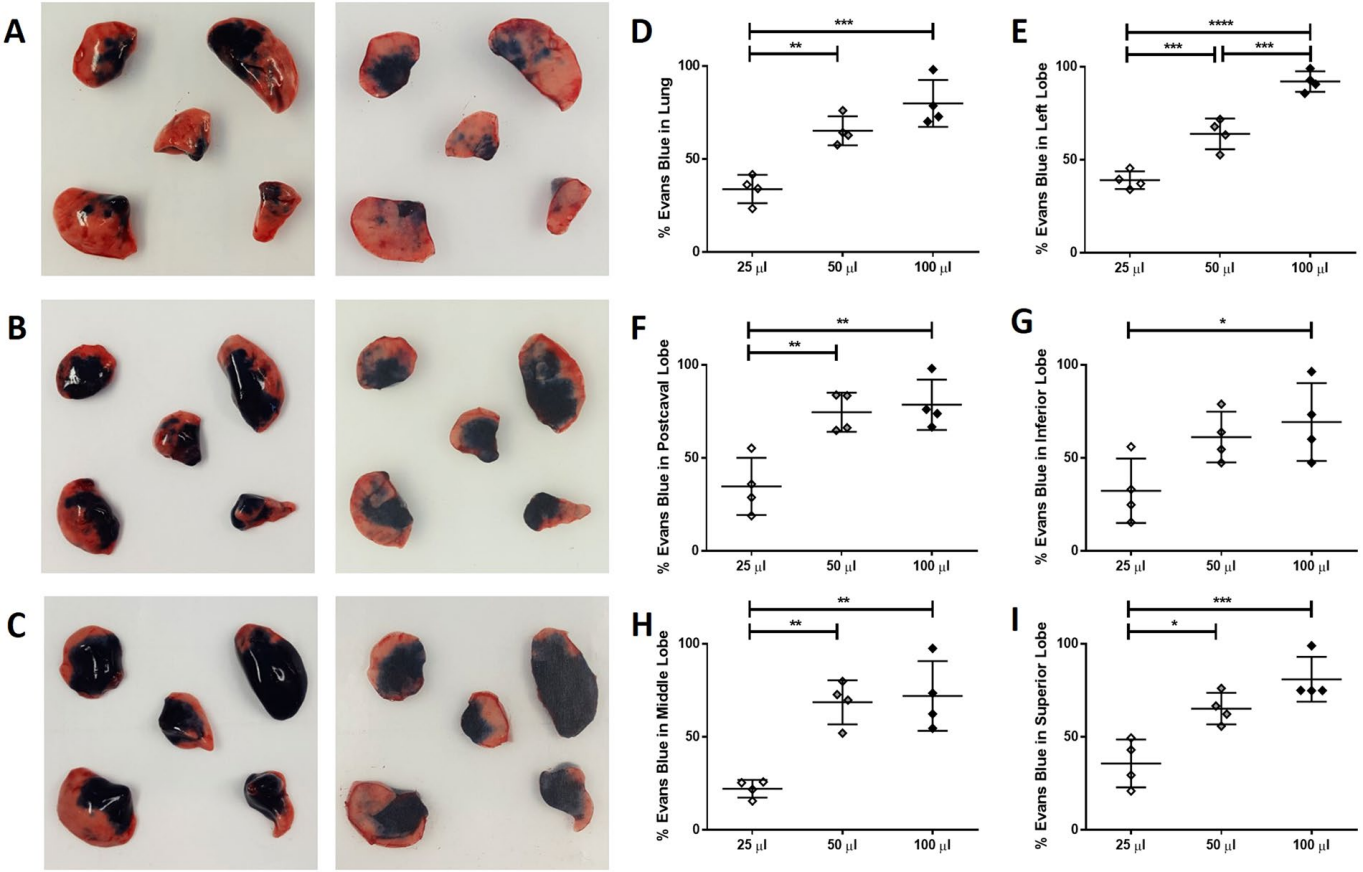
Effect of instillation volume on dispersal of Evans Blue dye in the lung. Male and female 8 to 12-week-old C57BL/6J mice were given one TO instillation of 25 (n = 4), 50 (n = 4) or 100 (n = 4) μl of PBS containing 0.15 mg of Evans Blue dye. Lungs were removed and photographed after 45 minutes. (**A, B, C**) Representative top (left column) and bottom (right column) views of the five lobes. In each image superior lobe (top left), left lobe (top right), postcaval lobe (bottom right) inferior lobe (bottom left), and middle lobe (center), are shown for (**A**) 25 μl, (**B**) 50 μl, and (**C**) 100 μl instillations. The percent area of each lung lobe stained with dye was calculated for both the top and bottom view using ImageJ and averaged as shown for (**D**) whole lung, (**E**) left lobe, (**F**) postcaval lobe, (**G**) inferior lobe, (**H**) superior lobe, and (**I**) middle lobe. Results were expressed as mean ± SEM. A one-way ANOVA was used to determine statistical significance between instillation volumes, 25 μl (white diamond), 50 μl (grey diamond), and 100 μl (black diamond) which are represented as (****p < 0.0001), (***p < 0.001), (**p < 0.01), and (*p < 0.05).

The effect of different volumes on the distribution of TO instilled material in the lungs was initially investigated using Evans Blue dye. The distribution of dye in the lung and all lobes of B6 mice was significantly greater for 50 and 100 μl instillations compared to the 25 μl volume (Fig. 1A–H). In contrast, although comparison of 50 and 100 μl instillations also revealed a significant difference, this effect was limited to the left lung lobe only (Fig. 1D–I). There were no differences in dispersal between males and females within each instillation volume (data not shown). This suggests that dispersal of the largest (i.e. 100 μl) volume is only effective in the largest lung lobe.

### Pulmonary dispersion of silica in 25 vs 50 μl instillations

**Figure 2:**
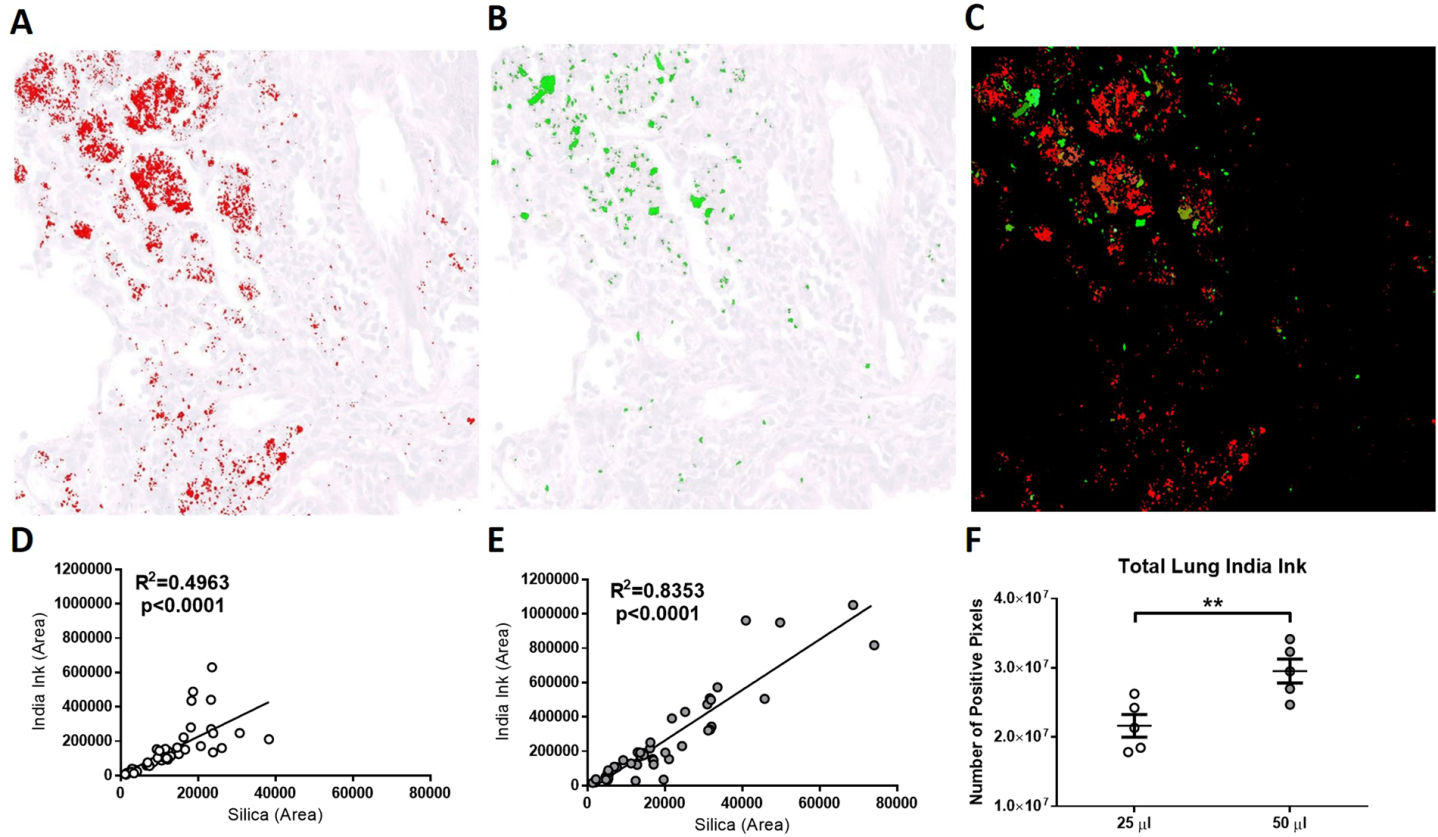
Effect of volume on silica and India ink dispersal in the lung. Female 8 to 12-week-old C57BL/6J mice were given one TO instillation of 10 mg of crystalline silica in a 20% India ink solution of 25 (n = 5) or 50 μl (n = 5). Images of the dispersed (**A**) India ink (red) and (**B**) silica (green) were overlaid on H&E stained sections. (**C**) Merged images of the India ink (red) and silica (green). Nine images per animal were taken to determine the correlation between India ink and silica in (**D**) 25 μl and (**E**) 50 μl volumes. (**F**) The amount of India ink in the entire lung at 40X was quantified. Because the 50ul group received twice the amount of India ink, their values were divided by two. Results were expressed as mean ± SEM. Statistical significance between 25 μl (white circle) and 50 μl (grey circle) instillations are represented as (**p < 0.01).

The increased dispersal of Evans Blue within the lung following 50 μl instillation suggested that larger volumes allowed greater dispersal of particulate material, therefore, subsequent experiments using crystalline silica compared 25 and 50 μl volumes. To test this hypothesis, 10 mg of silica was administered in 25, 50, or 100 μl of 20% India ink to visualize the dispersion, however 80% (4/5) of the mice receiving the 100 μl instillation survived less than 2 hours following exposure and were not included in this study. After one week, the total amount of India ink quantified in the lung was found to be significantly higher in the 50 μl group (Fig. 2F). Importantly, India ink and silica could be seen to clearly localize to areas of inflammation (Fig. 2A–C). Indeed, a positive correlation between the amount of silica and the amount of India ink was found with the 25 μl group (Fig. 2D) but was particularly strong in the 50 μl group (Fig. 2E). Taken together, the data show that the total amount of silica in the lung of mice receiving 50 μl is higher than the mice receiving 25 μl.

### Effect of instillation volume on silica-induced lung pathology

**Figure 3:**
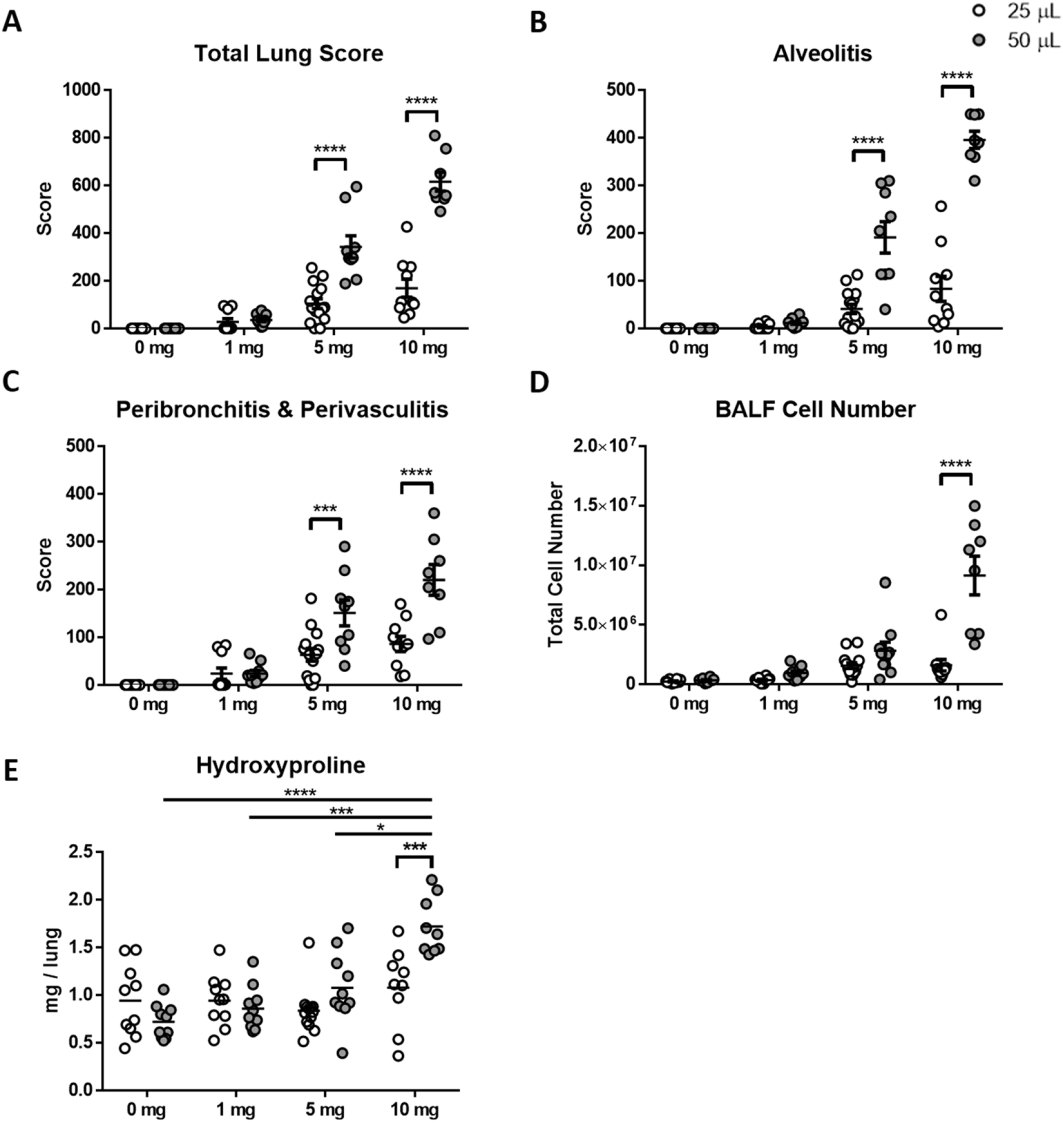
The effects of dose and volume of silica suspension on the severity of silicosis. Female 8 to 12-week-old C57BL/6J mice were given one TO instillation of 25 μl of PBS alone (n = 10) and with 1 (n = 10), 5 (n = 15), and 10 (n = 10) mg, or 50 μl of PBS alone (n = 10) and with 1 (n = 10), 5 (n = 10), and 10 (n = 8) mg crystalline silica for 4 weeks. Lungs were removed, and histology was scored for (**A**) total lung score, (**B**) alveolitis, and (**C**) peribronchitis and perivasculitis. Bronchoalveolar lavage fluid (BALF) was collected and cells were counted for (**D**) BALF cell numbers. Lung hydrolysates were used to measure (**E**) total lung hydroxyproline. A two-way ANOVA was used to determine statistical significance between dose and volume, 25 μl (white circle) and 50 μl (grey circle) instillations which are represented as (****p < 0.0001) and (***p < 0.001).

To determine whether the volume of transoral instillation has any effect on the development of silicosis, we exposed B6 mice to silica in either 25 or 50 μls and compared their lung pathology and inflammatory response. As expected, greater lung pathology was seen with higher silica doses for both instillation volumes (Fig. 3A–D, Supplemental Table S1, Supplemental Figs S1–2). However, instillation volume had a greater effect on total lung score, alveolitis, perivasculitis, and bronchoalveolar lavage fluid (BALF) cell number particularly at the higher exposures (Fig. 3A–D; p < 0.0001). Notably, there was a marked increase in severity between the 1 to 10 mg exposures with the 50 μl volume that was much less apparent with 25 μl. However, the 10 mg dose in a 50ul instillation proved toxic with 20% (2/10) mortality within the first 4 days of exposure.

To quantify the amount of silica-induced fibrosis, we measured total lung hydroxyproline content as a measure of collagen deposition. We observed no differences in the amount of hydroxyproline in mice receiving the 25 μl instillation, while mice receiving the 50 μl instillation had significantly higher hydroxyproline at 10 mg (Fig. 3E). Furthermore, there was a significant difference in the amount of total lung hydroxyproline in mice receiving 10 mg of silica between the 25 μl and 50 μl instillations (Fig. 3E). Thus, the volume of instillation plays a major factor in the induction and severity of silicosis pathology.

### Sex Effects in the development of silicosis

**Figure 4:**
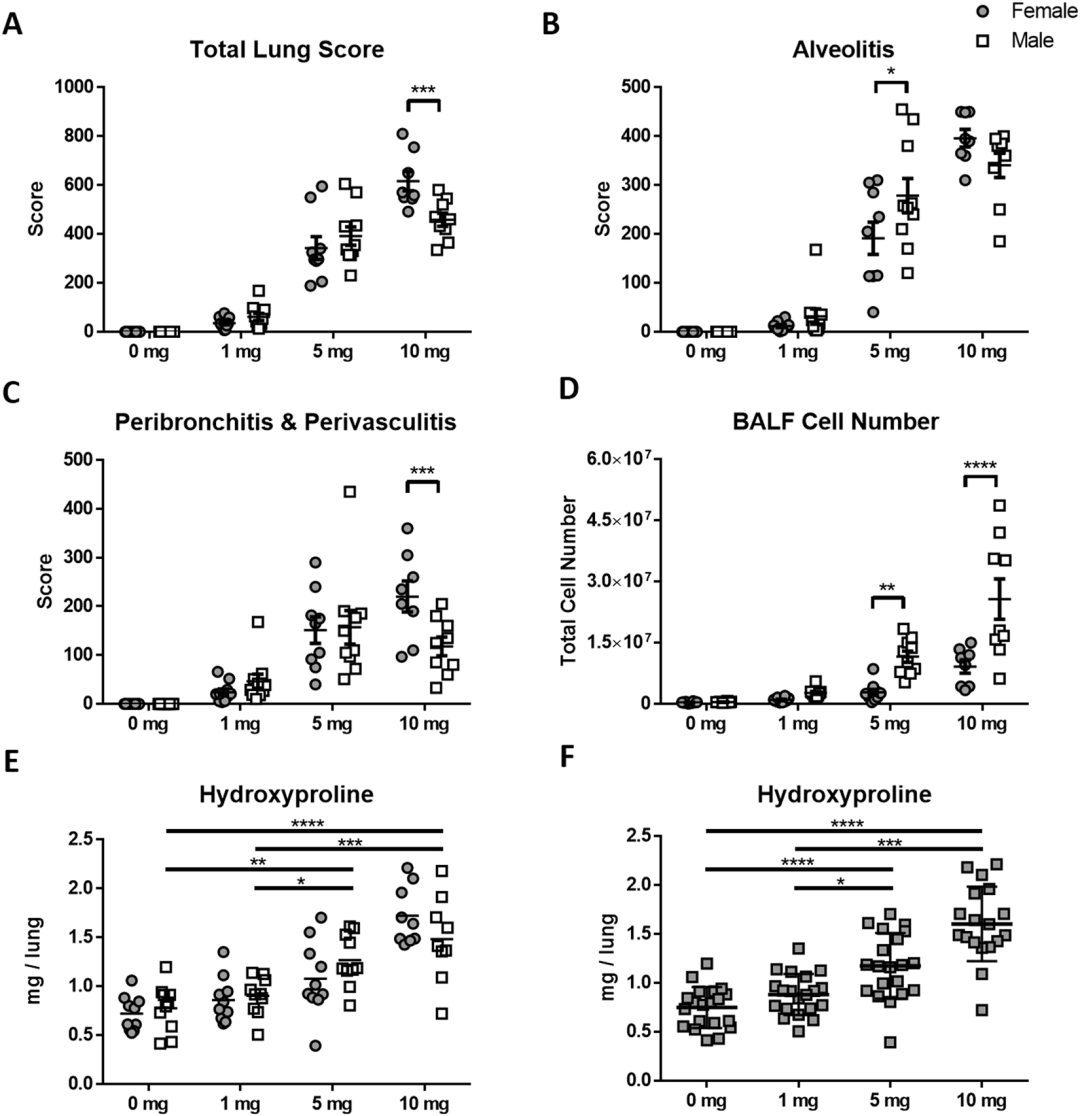
Sex effects in the development of silicosis. Female and male 6–8-week-old C57BL/6J mice were given one transoral instillation of 50 μl of PBS alone (n = 10) and with 1 (n = 10), 5 (n = 10), and 10 (n = 8–9) mg crystalline silica for 4 weeks. Lungs were removed, and histology was scored for (**A**) total lung score, (**B**) alveolitis, and (**C**) peribronchitis and perivasculitis. Bronchoalveolar lavage fluid (BALF) was collected and cells were counted for (**D**) BALF cell numbers. Lung hydrolysates were used to measure (**E**) total lung hydroxyproline with males and females separate and (**F**) together. Results were expressed as mean ± SEM. A two-way ANOVA was used to determine statistical significance between females (grey circle) and males (white square) which are represented as (****p < 0.0001), (***p < 0.001), and (*p < 0.05).

We^11^ and others^33–35^ have found differences in the responses of male and female mice to silica. To determine if silicosis following silica administration in a 50 μl volume exhibits sex effects male and female B6 mice were exposed to 1, 5, or 10 mg of crystalline silica in a volume of 50 μl of PBS for 4 weeks; controls were given PBS alone. Similar to the females, 10% (1/10) of males receiving 10 mg in 50 ul died within 10 days following instillation. We found a significant dose (p < 0.0001) effect for total lung score, alveolitis, perivasculitis, and BALF cell number for both sexes (Supplemental Table S1). Total lung score and perivasculitis and peribronchitis was significantly higher in females compared to males at the 10 mg dose. In contrast, male mice showed a significant increase in alveolitis at 5 mg (Fig. 4A–C, Supplemental Fig. S2) and BALF cell numbers were significantly increased in males compared to females at both 5 and 10 mg expsoures (Fig. 4D). We observed no significant differences in total lung hydroxyproline between males and females (Fig. 4E), however while females showed significantly higher hydroxyproline at 10 mg compared to 0 mg, 1 mg, and 5 mg (Fig. 3E), males showed significantly higher hydroxyproline at both 5 mg and 10 mg compared to 0 mg and 1 mg (Fig. 4E). Furthermore, when males and females are combined, there is no significant difference in the amout of hydroxyproline between 5 mg and 10 mg (Fig. 4F). Thus the more pronounced severity of silicosis produced using a 50 μl volume is assocaited with sex differences in response.

### Effects of instillation volume on silicosis in outbred mice

**Figure 5:**
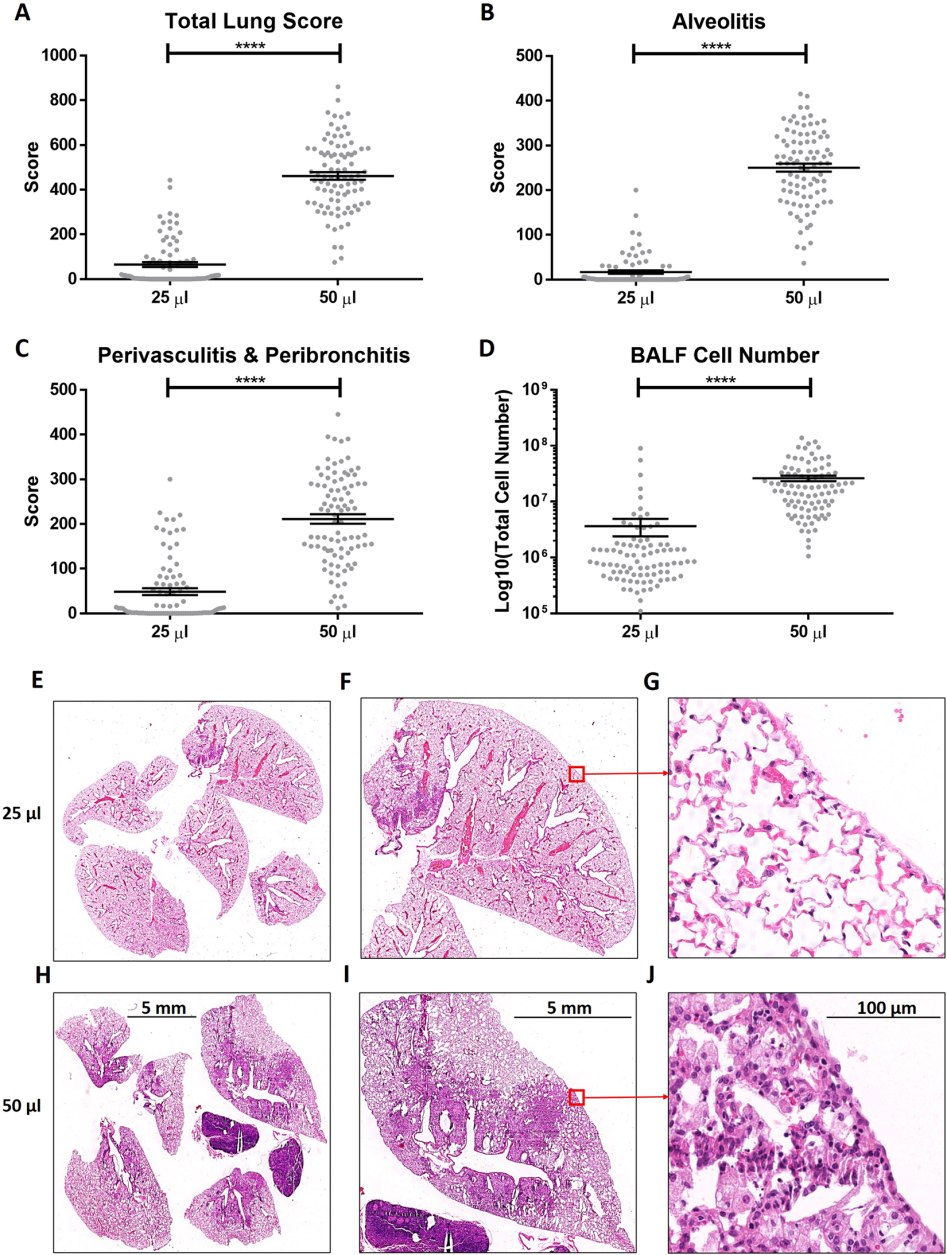
Effect of instillation volume on silicosis in outbred mice. Female and male 8 to 12-week-old DO mice were given one TO instillation of 5 mg crystalline silica in 25 (n = 86) or 50 μl (n = 89) of PBS. After 12 weeks lungs were removed, and histology was scored for (**A**) total lung score, (**B**) alveolitis, and (**C**) peribronchitis and perivasculitis. Bronchoalveolar lavage fluid (BALF) was collected and cells were counted for (**D**) BALF cell numbers. H&E images of the the whole lung for (**E**) 25 μl and (**H**) 50 μl at 0.5X, left lobe for (**F**) 25 μl and (**I**) 50 μl at 1X, and at 40X for (**G**) 25 μl and (**J**) 50 μl. Results expressed as mean ± SEM. A two-way ANOVA was used to determine statistical significance which are represented as (****p < 0.0001).

The inbred B6 was selected for initial experiments because it is susceptible to silicosis and is the most common strain studied (Supplementary Table S1)^9^. However, susceptibility to silicosis is significantly influenced by genetic background^9–11^. In previous studies using the DO mouse as a model of silicosis, mice were exposed to silica for 12 weeks following one 5 mg of silica in 25 μl transoral instillation^11^. To assess how the volume of instillation affects the severity of silicosis in mice of diverse genetic backgrounds, we compared silicosis in DO mice that received 5 mg of crystalline silica in 25 μl^11^ with those receiving 50 μl by TO instillation. The DO mice were exposed to silica for 12 weeks rather than the 4-week exposure in the B6 mice, Silicosis is a chronic inflammatory fibrotic disease and while the pathology, total lung score, alveolitis, perivasculitis and peribronchitis, and BALF cell numbers were all higher at 12 weeks as compared to 4 weeks (data not shown), all measurements of pathology (Fig. 5A–D) were significantly increased with the larger instillation volume. Importantly, the larger volume resulted in development of some degree of silicosis in essentially all mice, while a significant number of the animals given silica in the smaller volume failed to develop histological evidence of inflammation.

## Discussion

The observations made in this study are significant because there is no standardized method for crystalline silica exposure in experimental animals. A survey of the literature using TO, IT, or IN instillation methods (Supplemental Table S2) shows widely differing doses, volumes and exposure periods used in mouse models of silicosis. A recent study has even argued that the well-established intratracheal instillation method has yet to be standardized^36^. Studies have demonstrated that the severity of silica-induced inflammation and silicosis depend on the type and amount of silica instilled, the method of instillation, the length of exposure, and the mouse strain used. In this study we further establish the critical importance of volume in TO aspiration and its impact on the severity of alveolitis, perivasculitis and peribronchitis, and numbers of inflammatory infiltrating BALF cells. Instillation of Evans Blue dye showed this is due to greater dispersion of instilled material throughout the lung. This was confirmed by comparison of dispersal of India ink and crystalline silica. Significantly, the importance of instillation volume on disease severity was also demonstrated in genetically heterogeneous DO mice. These studies argue that optimizing instillation volume allows better distribution of crystalline silica and greater incidence of silicosis independent of genetic background.

Previous studies of instillation volume of soluble and particulate materials into the lung have focused on dispersal of soluble dyes, inks, and radiolabeled compounds. Pellikan black ink was used to determine the effect of instillation volume on lung distribution in hamsters^37^ while India ink was used to examine distribution in rats by IT administration under a variety of conditions^36^. Evans Blue dye has been used to visualize distribution in the lung by IT instillation and TO aspiration of crystalline silica^15^ and to ensure distribution of soluble recombinant antibodies into the lung in a model of mucosal allergic inflammation^38^. Radiolabeled sulfur colloid has been used to compare aspiration and instillation methods^31^ as well as different volumes^39^. In the current study, we investigated the dispersion of silica with two different instillation volumes of silica in an India ink solution. The amount of silica and India ink from different areas within the left lobe had a better correlation in the 50 μl volume compared to the 25 μl volume. Digital images of unstained sections were then used to quantify the amount of India ink dispersed across all lobes within that section. Similarly, to the Evans Blue dispersal, the amount of India ink quantified in the 50 μl instillation was higher than the 25 μl instillation. This raises the question of where the ink or silica goes in the 25 μl instillation if not in the lung. No staining was observed in the gut, therefore, we assume that a large portion of the silica and India ink does not reach the lung and remains largely within the upper airway and is eventually cleared as previously reported^40^. This is further supported by data obtained in the India ink quantification in the left lobe where we observe a much larger portion in the perihilar region in the 50 μl instillations compared to the 25 μl instillations (Supplemental Fig. S3). The efficient dispersal of Evans Blue in all lung lobes, and the better correlation between silica and India ink dispersal, shows that a single, appropriate volume results in effective dispersal of soluble and particulate material.

In addition to dispersal, several studies have also examined the effects of instillation volumes on consequent lung pathology with other agents and in other species. Introduction of silica into the lungs of experimental animals is achieved by constant inhalation or one or a few aspirations by IN, IT, or TO instillation^14^. TO aspiration was chosen for this study primarily because it was shown to distribute silica more evenly and uniformly with greater severity than the more frequently used IT instillation^14,15^. Similarly, instillation volume is known to impact experimental observations, but in reference to different species that vary in size^28^. We are unaware of any studies that examine the effect of the volume of the vehicle containing silica on its dispersal within the lung, development of lung inflammation, and silicosis, especially in outbred mice. Previous recommendations for instillation volumes were also for substances other than silica^28,32,37^. Notably, the IN instillation of radiolabeled sulfide colloid (0.2–0.8 μm) showed that volumes of ≥35 μl gave optimal delivery to the lower respiratory tract^39^. Comparison of 25 or 50 μl volumes of radiolabeled submicrometer sulfide colloid suggested that the larger volume delivered a greater amount to the lung, however, this was not formally documented^31^. In another study, a greater portion of radiolabeled 7 μm diameter microspheres, instilled IN into mice, entered the lungs when given in a 50 μl volume, whereas more particles remained in the nasal passages when a 10 μl volume was given^40^. With instillation of fluorescent Cy5.5 conjugated to dextran, 3 μl per gram of weight (approximately 30–75 μl/mouse) was the optimal volume with IT instillation^32^. Our findings show that instillation volume not only affects the distribution of micrometer sized crystalline silica but also the subsequent incidence and severity of inflammation.

In this study we found that both the dose and volume of silica administered affect the severity of disease. As expected, we found the severity of silicosis to be dose dependent, however 1 mg was insufficient to induce silicosis at either volume of instillation. This could be due to insufficient amounts of silica in the lung since a previous study found that approximately 20% of silica is retained in the lung following transoral instillation^41^. There were few differences found between controls and 1 or 5 mg using the 25 μl instillations. Conversely, using the same dose but a higher instillation volume of 50 μl, significant differences were found between controls and 5 and 10 mg in total lung pathology as well as alveolitis and perivasculitis and peribronchitis. A hydroxyproline assay was used to measure the amount of collagen in the lung. No differences were found in the amount of hydroxyproline between doses at the 25 μl instillations, however elevated levels were found in the higher instillation volume, with significantly higher levels at the 50 μl instillations in females receiving 10 mg and males receiving 5 and 10 mg. While no differences were found between the sexes, volume of instillation played a role in the amount of hydroxyproline in the lung with significantly higher levels found in females receiving 10 mg in 50 μl compared to 25 μl instillations. We found a significant sex affect in the number of BALF cells, with males accumulating much higher numbers of cells in the lung than females. However, at high doses females developed worse pathology than males. This supports our previous finding in the diversity outbred mouse, where we showed the males had higher BALF cell numbers than females^11^. One other study found gender effects in BALF cell numbers and hydroxyproline levels inconsistent with this study, however differences in the method of instillation and length of exposure might explain these discrepancies. Using a higher volume of instillation allows us to use a lower dose of silica and achieve the same, if not more severity of silicosis in our mouse models.

The severity of silica-induced pulmonary inflammation differs among inbred mouse strains^9,10^, suggesting genetic regulation. Using inbred C57BL/6 mice our findings show that optimizing the volume used to administer silica significantly increases the incidence and severity of lung pathology in both male and female mice. To examine if the administered volume affects disease severity in genetically diverse animals we compared responses in DO mice given 5 mg of crystalline silica in 25^11^ or 50 μl (this study). As found for the B6, the larger volume led to more severe pathology with essentially all mice developing disease to some extent, unlike mice that received 25 μls. Based on our results comparing Evans blue, India ink, and silica deposition, the difference between 25 and 50 μl is insufficient distribution of the silica into the lung parenchyma. Thus, the use of the appropriate instillation volume is critical for obtaining consistent silicosis in mice using the TO method and is essential for studying the influence of genetic variations such as in the DO mouse.

In this study we establish the critical importance of volume of transoral instillation for induction of experimental silicosis. Instilled volume had significant effects on the severity of alveolitis, perivasculitis and peribronchitis, and numbers of inflammatory infiltrating BALF cells. Thus, the appropriate instillation volume is necessary for obtaining consistent silicosis in mice using the TO method and is essential for studying the influence of genetic variations such as DO mouse studies.

## Methods

### Mice

C57BL/6Scr (B6) mice were obtained from The Scripps Research Institute (TSRI) Animal Colony. Diversity Outbred (DO) mice were obtained from Jackson Laboratory (Bar Harbor, ME). All mice were maintained under specific pathogen-free conditions. Animal rooms were kept at 68–72 °F and 60–70% humidity, with a 12 hr/12 hr light-dark cycle, and sterilized cages were replaced each week with fresh water and food (autoclaved standard grain diet 7012, Teklad, Envigo, Madison, WI, USA) to which the mice had access ad libitum. Experiments were initiated in 8–12-week-old animals. All animal procedures were carried out in accordance with the regulations and guidelines of the Institutional Animal Care and Use Committee at The Scripps Research Institute. The experimental protocols were approved by the Institutional Animal Care and Use Committee at The Scripps Research Institute (protocol# 08-0150) and use of silica was approved by TSRI Department of Environmental Health and Safety.

### Evans Blue instillation

To visualize the effect of volume on dispersal of aspirated fluid, 0.15 mg of Evans Blue dye (Fisher Scientific Company, Fair Lawn, New Jersey) in PBS was administered TO in 25, 50 and 100 μl volumes (0.6, 0.3, and 0.15% solutions) to male (n = 2) and female (n = 2) B6 mice. Lungs were recovered 45 minutes after instillation, separated into lobes and photographed (top and bottom view). ImageJ (https://imagej.nih.gov/ij/) was used to determine area/lung lobe and the portion stained with dye calculated as a percent of the total area/lobe and as a percent of the total area of all five lobes/mouse as an average of both the top and bottom view images.

### Exposure to crystalline silica

Crystalline silica (Min-U-Sil-5, average particle size 1.5–2 μm; U.S. Silica Company, Frederick, MD) was washed in 1 M HCl at 100 °C for 2 h, washed three times with sterile water, autoclaved for 1 h at 121 °C, and dried and reconstituted in PBS^42^. Immediately prior to use, silica was disbursed by sonication. Female and male mice were exposed to a single dose of 1, 5, or 10 mg by TO instillation in a volume of 25 μl or 50 μl in PBS as described^16,43^. Briefly, mice were anesthetized with isoflurane and suspended on an angled board by a rubber band hooking the front teeth after which the tongue was pulled forward and sideways (to block the swallow reflex). Silica solution was then delivered by micropipette to the posterior pharynx where it was spontaneously aspirated into the lungs.

### Bronchoalveolar lavage and lung histology

Mice were sacrificed at 4- or 12-weeks post-exposure and bronchoalveolar lavage fluid (BALF) was collected. Total cell counts were determined using a Countess II FL Automated Cell Counter (Thermo Fisher Scientific, Waltham, MA). Lungs were excised and fixed for 24 hours in zinc formalin. Hematoxylin and eosin paraffin sections (5 μm) were prepared by TSRI Histology Core and slides scanned by Digital Image Hub (Slidepath, Dublin, Ireland). Silicosis was scored under blinded conditions for the percent of each lobe affected by alveolitis or peribronchitis/perivasculitis (5 lobes, 500 maximum score each)^11^. The two values were combined to give a total lung score.

### Detection and quantitation of pulmonary silica and India ink

To visualize the dispersion of silica, a cohort of female B6 mice were exposed to 10 mg of silica in 25, 50, or 100 μl in 20% India ink solution for 1 week. To detect India ink and silica in the lung, unstained lung sections of identical areas of the left lobe were analyzed with both brightfield microscopy and polarizing lenses, respectively. Nine areas representing three each for perihilar, mid and distal lung regions were analyzed (Supplemental Fig. S3). Silica and India ink were detected and quantified by setting the threshold and region of interest (ROI) in ImageJ to detect birefringence (silica) or black color (India ink), respectively. The total amount of India ink in the lung section was determined by ImageScope at 40X using a positive pixel count algorithm. Both 25 and 50 μl instillations were composed of a 20% India ink solution for visualization, therefore the 50 μl group received twice as much India ink. This was corrected for by dividing the total amount of India ink by two for the 50ul group in the comparison of total lung India ink.

### Hydroxyproline analysis

Briefly, formalin fixed paraffin embedded whole lungs were acid hydrolyzed in 0.8 ml 6 N HCl at 95 °C for 20 h. Samples were centrifuged for 10 min at 13,000 rpm, and hydroxyproline residue quantification was performed on the resulting supernatant at a 1:20 dilution. Hydroxyproline was determined using the Quickzyme Sensitive Tissue Collagen Assay (QZBtiscol1, QuickZyme, Leiden, Netherlands) as described by the manufacturer.

### Statistical analysis

Data are expressed as mean and standard error unless otherwise stated. Statistical analysis used GraphPad Software V6 (San Diego, CA). One-and two-way ANOVA were used for multiple comparisons and Pearson’s correlation coefficient for linear relationships. p < 0.05 was considered significant.

### Ethics approval and consent to participate

All animal procedures were approved by TSRI Institutional Animal Care and Use Committee (IACUC).

## Supplementary information

Supplemental Materials

## Data Availability

The datasets used and/or analyzed during the current study are available from the corresponding author on reasonable request.

## References

[1] Leung CC, Yu IT, Chen W (2012). Silicosis. Lancet.

[2] Pollard KM (2016). Silica, Silicosis, and Autoimmunity. Front Immunol.

[3] Kawasaki H (2015). A mechanistic review of silica-induced inhalation toxicity. Inhal Toxicol.

[4] Steenland K, Brown D (1995). Mortality study of gold miners exposed to silica and nonasbestiform amphibole minerals: an update with 14 more years of follow-up. American journal of industrial medicine.

[5] Dosemeci M (1994). Indirect validation of a retrospective method of exposure assessment used in a nested case-control study of lung cancer and silica exposure. Occup Environ Med.

[6] Rice C, Jin N, Cocco P, Dosemeci M, Buncher CR (2011). The exposure metric: does including time since exposure in the calculation of working lifetime exposure provide a better understanding of disease risk than the cumulative exposure?. Med Lav.

[7] Hnizdo E, Sluis-Cremer GK (1993). Risk of silicosis in a cohort of white South African gold miners. American journal of industrial medicine.

[8] Buchanan D, Miller BG, Soutar CA (2003). Quantitative relations between exposure to respirable quartz and risk-of silicosis. Occupational and Environmental Medicine.

[9] Callis AH, Sohnle PG, Mandel GS, Wiessner J, Mandel NS (1985). Kinetics of inflammatory and fibrotic pulmonary changes in a murine model of silicosis. J Lab Clin Med.

[10] Davis GS, Leslie KO, Hemenway DR (1998). Silicosis in mice: effects of dose, time, and genetic strain. J Environ Pathol Toxicol Oncol.

[11] Mayeux JM (2018). Silicosis and Silica-Induced Autoimmunity in the Diversity Outbred Mouse. Front Immunol.

[12] Brown JM, Archer AJ, Pfau JC, Holian A (2003). Silica accelerated systemic autoimmune disease in lupus-prone New Zealand mixed mice. Clin Exp Immunol.

[13] Bates MA (2015). Silica Triggers Inflammation and Ectopic Lymphoid Neogenesis in the Lungs in Parallel with Accelerated Onset of Systemic Autoimmunity and Glomerulonephritis in the Lupus-Prone NZBWF1 Mouse. PLoS One.

[14] Moore BB (2013). Animal models of fibrotic lung disease. Am J Respir Cell Mol Biol.

[15] Lakatos HF (2006). Oropharyngeal aspiration of a silica suspension produces a superior model of silicosis in the mouse when compared to intratracheal instillation. Exp Lung Res.

[16] Lacher SE, Johnson C, Jessop F, Holian A, Migliaccio CT (2010). Murine pulmonary inflammation model: a comparative study of anesthesia and instillation methods. Inhal Toxicol.

[17] Thakur SA, Beamer CA, Migliaccio CT, Holian A (2009). Critical role of MARCO in crystalline silica-induced pulmonary inflammation. Toxicol Sci.

[18] Trentin PG (2015). Annexin A1 mimetic peptide controls the inflammatory and fibrotic effects of silica particles in mice. Br J Pharmacol.

[19] Bissonnette E, Rola-Pleszczynski M (1989). Pulmonary inflammation and fibrosis in a murine model of asbestosis and silicosis. Possible role of tumor necrosis factor. Inflammation.

[20] Huaux F (1999). Lung fibrosis induced by silica particles in NMRI mice is associated with an upregulation of the p40 subunit of interleukin-12 and Th-2 manifestations. Am J Respir Cell Mol Biol.

[21] Huaux F (2002). A profibrotic function of IL-12p40 in experimental pulmonary fibrosis. J Immunol.

[22] Bandeira E (2018). Therapeutic effects of adipose-tissue-derived mesenchymal stromal cells and their extracellular vesicles in experimental silicosis. Respir Res.

[23] Lopes-Pacheco M (2014). Infusion of bone marrow mononuclear cells reduces lung fibrosis but not inflammation in the late stages of murine silicosis. PLoS One.

[24] Kato K (2017). Muc1 deficiency exacerbates pulmonary fibrosis in a mouse model of silicosis. Biochem Biophys Res Commun.

[25] Karkale S, Khurana A, Saifi MA, Godugu C, Talla V (2018). Oropharyngeal administration of silica in Swiss mice: A robust and reproducible model of occupational pulmonary fibrosis. Pulm Pharmacol Ther.

[26] Misson P, van den Brule S, Barbarin V, Lison D, Huaux F (2004). Markers of macrophage differentiation in experimental silicosis. J Leukoc Biol.

[27] Linderholm AL, F. L, Bein KJ, Pinkerton KE, Last JA (2015). A quantitative comparison of intranasal and intratracheal administration of coarse PM in the mouse. Integr Pharm Toxicol Gentoxicol.

[28] Driscoll KE (2000). Intratracheal instillation as an exposure technique for the evaluation of respiratory tract toxicity: uses and limitations. Toxicol Sci.

[29] De Vooght V (2009). Oropharyngeal aspiration: an alternative route for challenging in a mouse model of chemical-induced asthma. Toxicology.

[30] Egger C (2013). Administration of bleomycin via the oropharyngeal aspiration route leads to sustained lung fibrosis in mice and rats as quantified by UTE-MRI and histology. PLoS One.

[31] Foster WM, Walters DM, Longphre M, Macri K, Miller LM (2001). Methodology for the measurement of mucociliary function in the mouse by scintigraphy. J Appl Physiol (1985).

[32] Helms, M. N., Torres-Gonzalez, E., Goodson, P. & Rojas, M. Direct tracheal instillation of solutes into mouse lung. *J Vis Exp*, 10.3791/1941 (2010). 10.3791/1941 PMC3156004 20834218

[33] Brass DM (2010). Gender influences the response to experimental silica-induced lung fibrosis in mice. Am J Physiol Lung Cell Mol Physiol.

[34] Pollard KM (2012). Gender differences in autoimmunity associated with exposure to environmental factors. J Autoimmun.

[35] Latoche JD (2016). Secreted Phosphoprotein 1 and Sex-Specific Differences in Silica-Induced Pulmonary Fibrosis in Mice. Environ Health Perspect.

[36] Hasegawa-Baba Y, Kubota H, Takata A, Miyagawa M (2014). Intratracheal instillation methods and the distribution of administered material in the lung of the rat. J Toxicol Pathol.

[37] Baxter, D. W. & Port, C. D. In *Experimental Lung Cancer* (eds Karbe, E. & Park, J. F.) 86–91 (Springer-Verlag Berlin, 1974).

[38] Keane-Myers AM, Gause WC, Finkelman FD, Xhou XD, Wills-Karp M (1998). Development of murine allergic asthma is dependent upon B7-2 costimulation. J Immunol.

[39] Southam DS, Dolovich M, O’Byrne PM, Inman MD (2002). Distribution of intranasal instillations in mice: effects of volume, time, body position, and anesthesia. Am J Physiol Lung Cell Mol Physiol.

[40] Eyles JE, Williamson ED, Alpar HO (1999). Immunological responses to nasal delivery of free and encapsulated tetanus toxoid: studies on the effect of vehicle volume. Int J Pharm.

[41] Barbarin V, Xing Z, Delos M, Lison D, Huaux F (2005). Pulmonary overexpression of IL-10 augments lung fibrosis and Th2 responses induced by silica particles. Am J Physiol Lung Cell Mol Physiol.

[42] Hamilton RF, Thakur SA, Mayfair JK, Holian A (2006). MARCO mediates silica uptake and toxicity in alveolar macrophages from C57BL/6 mice. J Biol Chem.

[43] Biswas R, Trout KL, Jessop F, Harkema JR, Holian A (2017). Imipramine blocks acute silicosis in a mouse model. Part Fibre Toxicol.

