# Supplementary material for: Development of experimental silicosis in inbred and outbred mice depends on instillation volume
Source: Sci Rep. 2019 Oct 2;9:14190. doi: 10.1038/s41598-019-50725-9 (PMC6775097; doi:10.1038/s41598-019-50725-9)
Supplement: Supplementary file 1 — Supplemental Materials [file 41598_2019_50725_MOESM1_ESM.docx]

**Development of experimental silicosis in inbred and outbred mice depends on instillation volume.**

Jessica M. Mayeux^1^, Dwight H. Kono^2^, and Kenneth Michael Pollard^1*^

^1^ Department of Molecular Medicine ^2^ Department of Immunology and Microbiology, The Scripps Research Institute, 10550 North Torrey Pines Road, La Jolla, CA 92037, USA

**SUPPLEMENTAL MATERIALS**


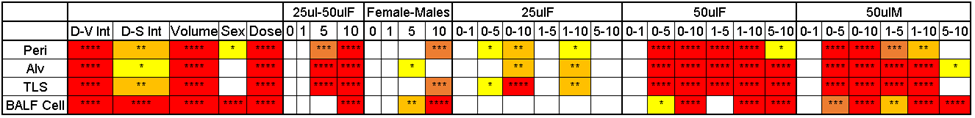


**Supplementary Table S1. Statistics for ANOVA analysis.** Female and male 8 to 12 week old C57BL/6J mice were given one TO instillation of 25 μl of PBS alone (n=10) and with 1 (n=10), 5 (n=15), and 10 (n=10) mg, or 50 μl of PBS alone (n=10) and with 1 (n=10), 5 (n=10), and 10 (n=8-9) mg crystalline silica for 4 weeks. Lungs were removed, and histology was scored for total lung score (TLS), alveolitis (Alv), and peribronchitis and perivasculitis (Peri). Bronchoalveolar lavage fluid (BALF) was collected and cells were counted for BALF cell numbers. 25 μl-50 μl two-way ANOVA results for dose and volume (D-V). Two-way ANOVA results. Female-Males two-way ANOVA results for dose and sex (D-S). 25 μl Females (25ulF), 50 μl Females (50ulF), 50 μl Males (50ulM) ordinary one-way ANOVA for dose effects. Statistical significance shown as (****p < 0.0001), (***p < 0.001), (**p<0.01), and (*p < 0.05).


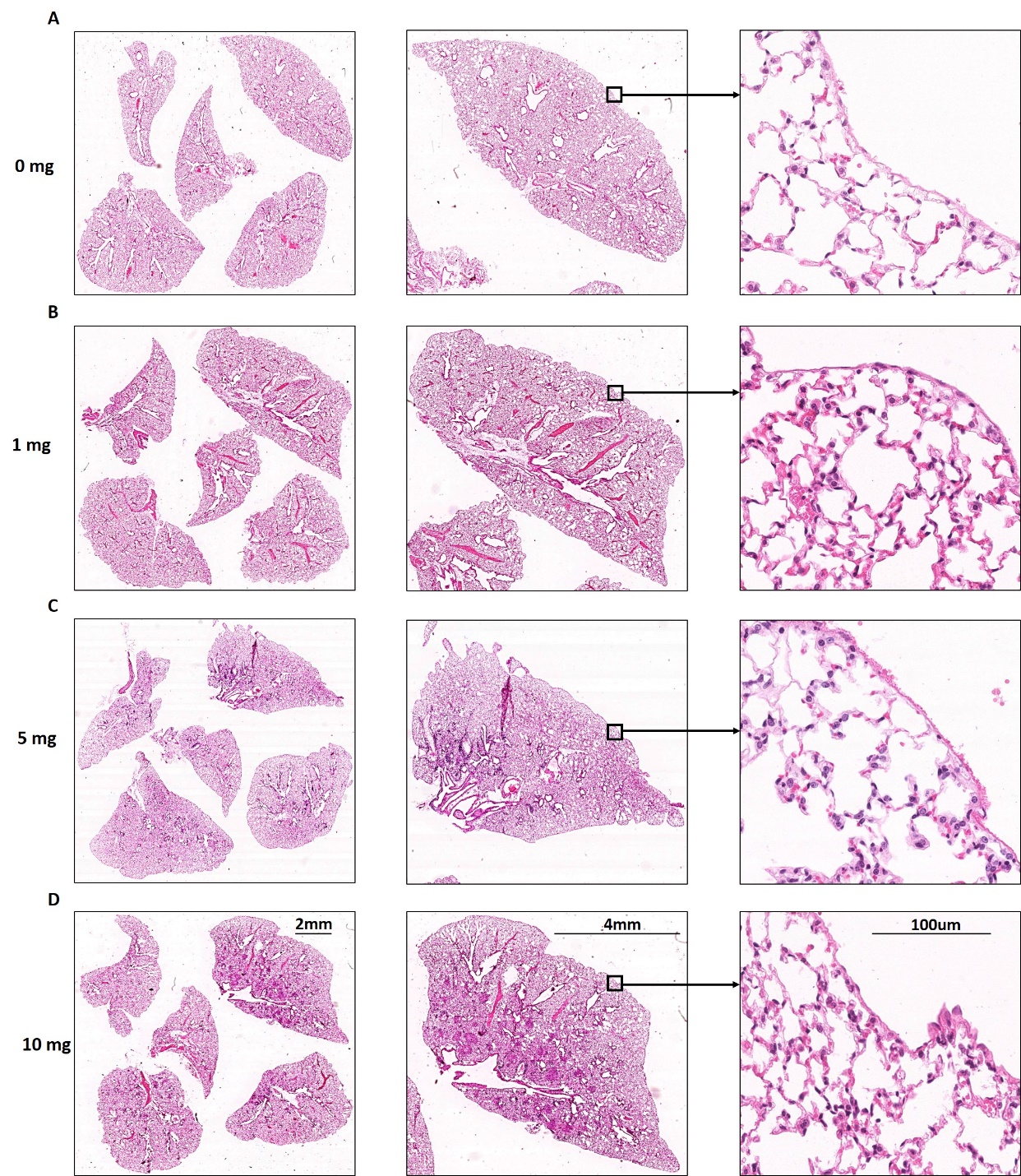


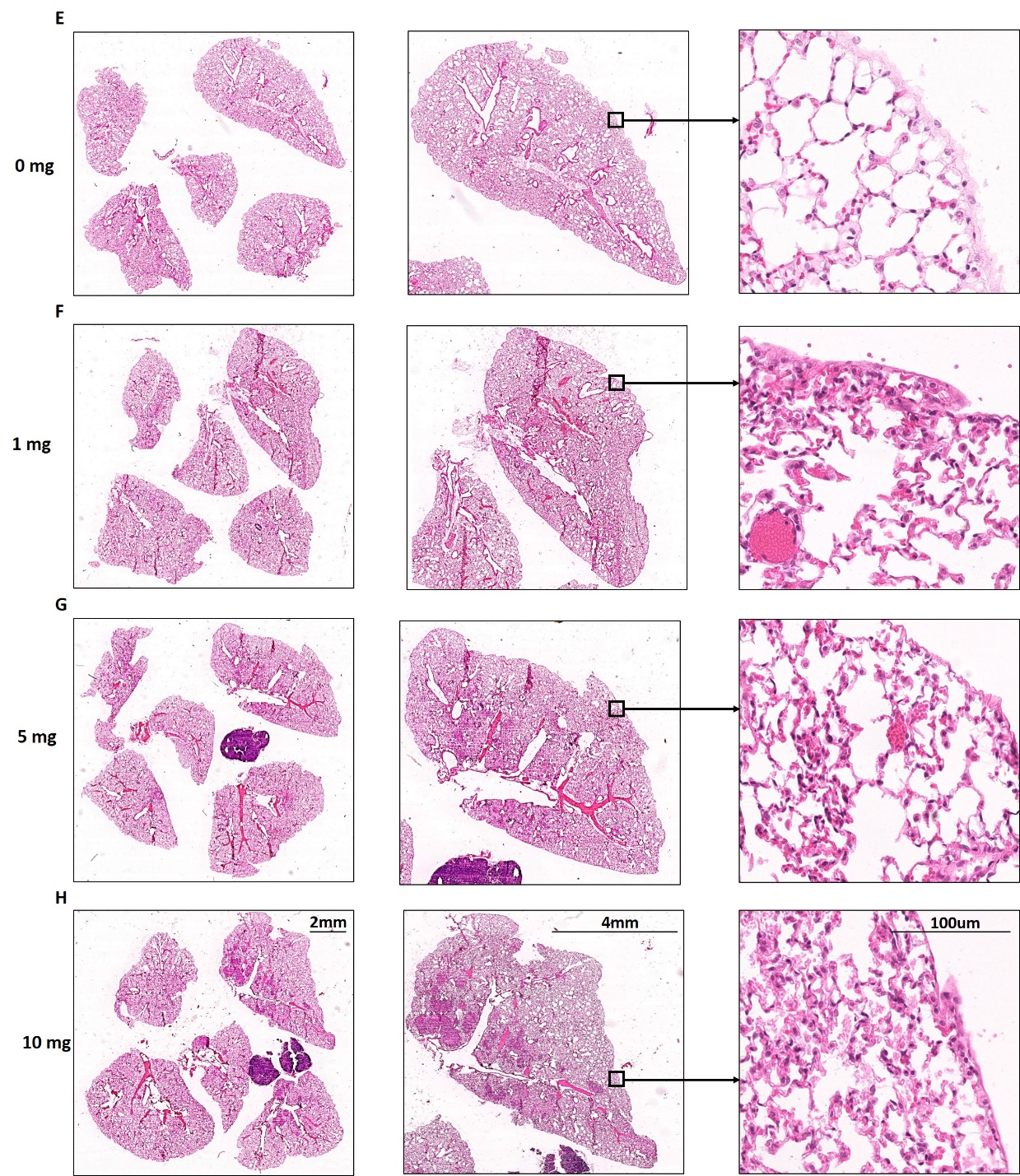


**Supplementary Figure S1. The effects of dose and volume of silica suspension on the severity of silicosis by lung lobe.** Female 8 to 12-week-old C57BL/6J mice were given one TO instillation of 25 μl of PBS alone (n=10) and with 1 (n=10), 5 (n=15), and 10 (n=10) mg, or 50 μl of PBS alone (n=10) and with 1 (n=10), 5 (n=10), and 10 (n=8) mg crystalline silica for 4 weeks. H&E images from mice with a total lung score representing the average of the group were chosen from 0mg A) 25 μl and E) 50 μl, 1mg B) 25ul and F) 50ul, 5mg C) 25 μl and G) 50 μl, and 10mg D) 25 μl and H) 50 μl. The 3 panels per treatment group show the whole lung at 0.5X (left panel), left lobe at 1X (middle panel), and at 40X (right panel).


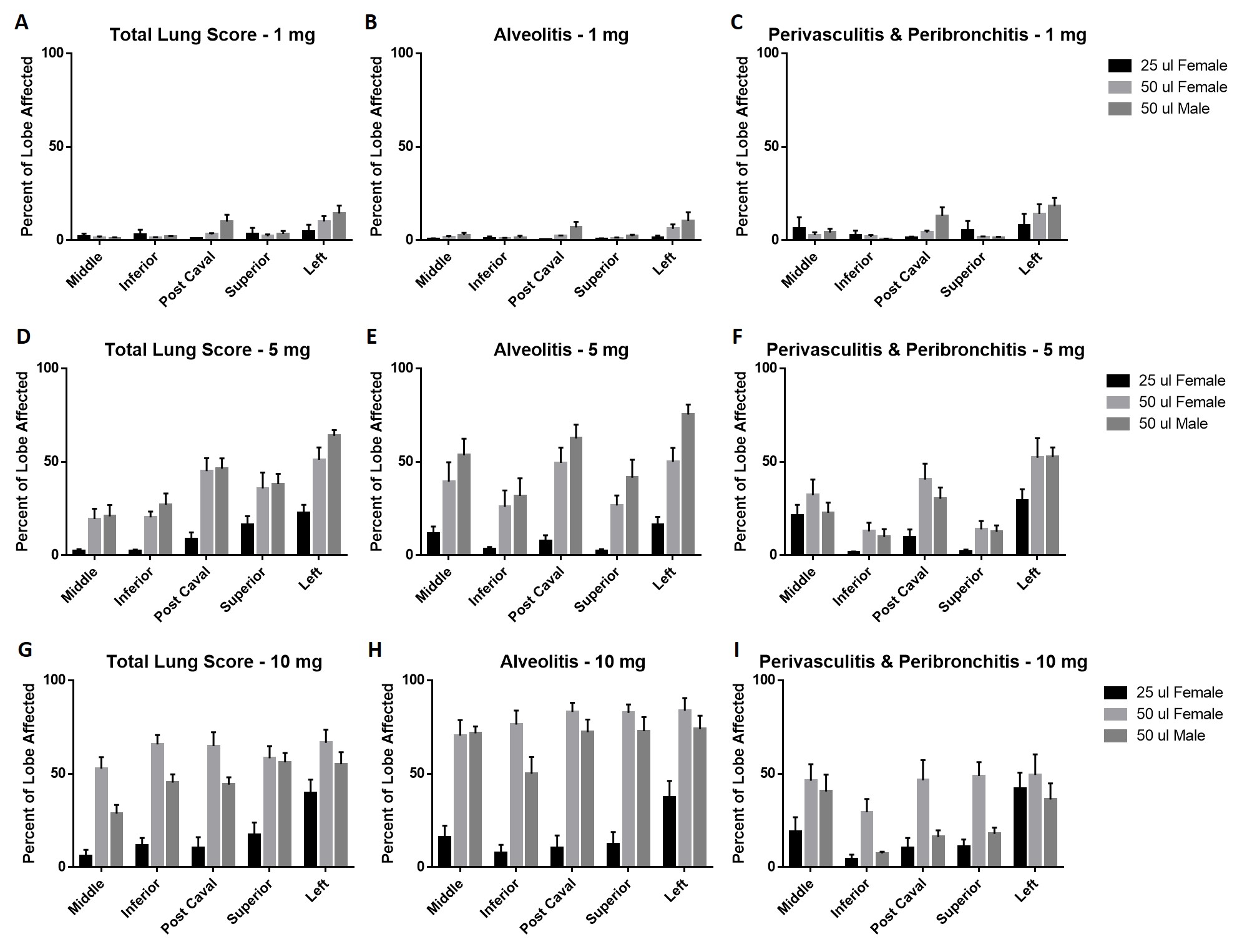


**Supplementary Figure S2.** **The effects of dose and volume of silica suspension on the severity of silicosis by lung lobe.** Female and male 8 to 12-week-old C57BL/6J mice were given one TO instillation of 25 μl of PBS alone (n=10) and with 1 (n=10), 5 (n=15), and 10 (n=10) mg, or 50 μl of PBS alone (n=10) and with 1 (n=10), 5 (n=10), and 10 (n=8-9) mg crystalline silica for 4 weeks. Lungs were removed, and H&E stained histology sections were scored for total lung score (A) 1 mg, (D) 5 mg, and (G) 10 mg, alveolitis (B) 1 mg, (E) 5 mg, and (H) 10 mg, and peribronchitis and perivasculitis (C) 1 mg, (F) 5 mg, and (I) 10 mg. The percent of lobe affected was calculated per lung lobe: middle, inferior, post caval, superior, and left lobe. Results were expressed as mean ± SEM.


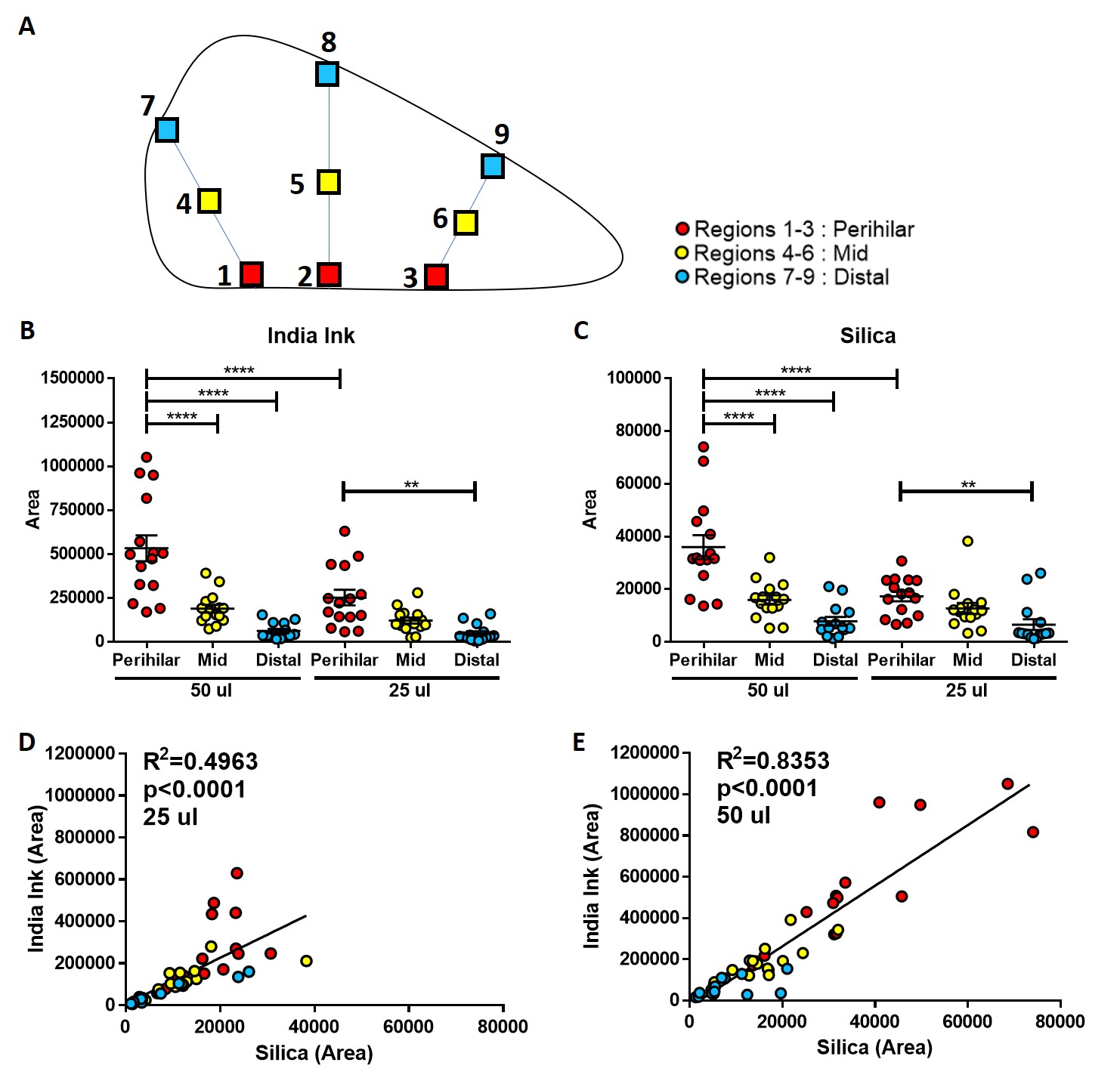


**Supplementary Figure S3. The effect of volume on silica and India ink dispersal in the left lobe.** Female 8 to 12-week-old C57BL/6J mice were given one TO instillation of 10 mg of crystalline silica in a 20% India Ink solution with either a 25 μl (n=5) or 50 μl (n=5) volume for one week. To detect India ink and silica in the lung, unstained lung sections of identical areas of the left lobe were analyzed with both brightfield microscopy and polarizing lenses, respectively. (A) Diagram of the nine areas representing three each for perihilar (regions 1-3, red), mid (regions 4-6, yellow) and distal (regions 7-9, blue) lung regions were analyzed.

Image J was used to quantify the area of (B) India ink (black color) and (C) Silica (birefringence). Correlation between the quantified area of India ink and silica in (D) 25 μl and (E) 50 μl volumes. Because the 50ul group received twice amount of India ink, their values were divided by two. Results were expressed as mean ± SEM. A one-way ANOVA was used to determine statistical significance between regions which are represented as (****p < 0.0001) and (**p<0.01).

| **Supplementary Table S2. Parameters used for induction of silicosis by different methods of silica instillation.** | | | | | | |
| --- | --- | --- | --- | --- | --- | --- |
|  |  |  |  |  |  |  |
| **Route of Administration** | **Silica** | **Strain** | **Volume** | **Dose** | **Exposure Period** | **Reference** |
| ***Oropharyngeal (OP)*** |  |  |  |  |  |  |
|  | DQ12*, d50**=2.2 μm | C57BL/6, female | 60 μl | 2.5 mg | 3-60 days | Misson P et al, 2004 |
|  | Cristobalite, mean size 0.8um (C & E Minerals, King of Prussia, PA) | C57BL/6, male | 40 μl | 2.0 mg | 21 days | Lakatos HF et al, 2006 |
|  | Min-U-Sil-5*** average particle size 1.5-2 μm (Pennsylvania Glass Sand Co, Pittsburgh, PA) | Balb/c, sex not stated | 25 μl | 1.0 mg | 3 days | Lacher SE et al, 2010 |
|  | Mean diameter 1-5 μm (source not stated) | C57BL/6, male | 40 μl | 5.0 mg | 3-21 | Kato K et al, 2017 |
|  | Min-U-Sil-5 (Sigma-Aldrich, USA) | Swiss Albino, male | 60 μl | 1.5 mg | 21 days | Karkale S et al, 2018 |
|  | Min-U-Sil-5, average particle size 1.5–2 µm (U.S. Silica Company, Frederick, MD, USA) | Diversity Outbred, male and female | 25 μl | 5.0, 10.0 mg | 84 days | Mayeux JM et al, 2018 |
|  |  |  |  |  |  |  |
| ***Intratracheal (IT)*** |  |  |  |  |  |  |
|  | Min-U-Sil-5 (Pennsylvania Glass Sand Co, Pittsburgh, PA) | Balb/c, sex not stated | 25 μl | 5.0 mg | 14-252 | Bissonnette E et al, 1989 |
|  | DQ12, d50=2.2 μm (Source not stated) | NMRI, female | 100 μl | 2.5 mg | 3-120 days | Huaux F et al, 1999 |
|  | (source not stated) | C57BL/6, female | 50 μl | 1.0-5.0 mg | 3-60 days | Huaux F et al, 2002 |
|  | Cristobalite, mean size 0.8um (C & E Minerals, King of Prussia, PA) | C57BL/6, male | 40 μl | 2.0 mg | 21 days | Lakatos HF et al, 2006 |
|  | Min-U-Sil-5 average particle size 1.5-2 μm (Pennsylvania Glass Sand Co, Pittsburgh, PA) | Balb/c, sex not stated | 25 μl | 1.0 mg | 3 days | Lacher SE et al, 2010 |
|  | 80% between 1 and 5 μm, (Sigma-Aldrich, St. Louis, MO) | C57BL/6, male and female | 50 μl | 10.0 mg | 70 days | Lopes-Pacheco M et al, 2014 |
|  | Min-U-Sil-5 (median aerodynamic diameter = 2.2 μm) | C57BL/6, male and female | 60ul | 0.2g/kg  (~4mg) | 14 days | Brass, D. M. et al, 2010  Latoche, J. D. et al., 2016 |
|  | (source not stated) | C57BL/6, female | 50 μl | 20.0 mg | 30 days | Bandeira E et al, 2018 |
|  |  |  |  |  |  |  |
| ***Intranasal (IN)*** |  |  |  |  |  |  |
|  | Min-U-Sil-5, average particle size 1.5–2 μm (Pennsylvania Glass Sand Co, Pittsburgh, PA) | C57BL/6, male and female | 25 μl | 1.0 mg | 3-28 days | Thakur SA et al, 2009 |
|  | Min-U-Sil-5 average particle size 1.5-2 μm (Pennsylvania Glass Sand Co, Pittsburgh, PA) | Balb/c, sex not stated | 25 μl | 1.0 mg | 3 days | Lacher SE et al, 2010 |
|  | 0.5 – 10 μm (Sigma Aldrich Corp, St Louis, MO, USA) | Swiss Webster, male | 50 μl | 10.0 mg | 7 days | Trentin PG et al, 2015 |
|  |  |  |  |  |  |  |

**Supplementary Table S2. Parameters used for induction of silicosis by different methods of silica instillation.**

* DQ 12 is a natural quartz from a geological source in Dörentrup, Germany. It is a quartz sand with a content of 87% crystalline silica.

** d50=2.2 μm means that 50% of the sample has a size of 2.2 μm or smaller.

*** Min-U-Sil is a trade name of a ground quartz dust sold by different companies. Min-U-Sil 5 is a notation that the particle size is ≤ 5 µm in diameter.

Source of * and ***, *Silica, Some Silicates, Coal Dust and para-Aramid Fibrils*. IARC Monographs on the Evaluation of Carcinogenic Risks to Humans, No. 68. IARC Working Group on the Evaluation of Carcinogenic Risk to Humans. Lyon (FR): International Agency for Research on Cancer; 1997.
